# The Knowledge, Attitudes, and Practices Regarding Diabetic Ketoacidosis Among Diabetic Patients in the Northern and Western Regions of Saudi Arabia

**DOI:** 10.7759/cureus.55617

**Published:** 2024-03-06

**Authors:** Abdelmuhsin Hassan, Atheer Alhuthaili, Moayad Mudawi, Mohamed Elamin, Tasneem H Atia, Mohammed Alshinqiti, Khaled Alfawaz, Mohammed Alamri, Albaraa Sufyani, Raghad M Alasmari, Njoud Alkedaisi, Essam Alhazmi

**Affiliations:** 1 Internal Medicine, College of Medicine, Hail University, Hail, SAU; 2 Faculty of Medicine, Hail University, Hail, SAU; 3 General Practice, Sudan Medical Council, Khartoum, SDN; 4 Medicine, Elrazi University, Khartoum, SDN; 5 Medicine and Surgery, Sulaiman Al Rajhi University, Bukayriyah, SAU; 6 Faculty of Medicine, King Abdulaziz University, Jeddah, SAU; 7 Faculty of Medicine, Taif University, Taif, SAU; 8 Faculty of Medicine, Umm Al-Qura University, Mecca, SAU; 9 Faculty of Medicine, Jazan University, Jazan, SAU

**Keywords:** type 2 diabetes mellitus (dm), insulin, glucose, glycated hemoglobin (hba1c), diabetic ketoacidosis (dka), type i diabetes mellitus

## Abstract

Background and objective

Diabetes mellitus (DM) is a chronic debilitating metabolic disease caused by insulin deficiency. Diabetic ketoacidosis (DKA) is a potentially fatal complication characterized by acute hyperglycemia and metabolic acidosis. In light of the high prevalence of DM in Saudi Arabia, we sought to investigate the knowledge, attitudes, and practices of the Saudi general population about DKA.

Methods

An online self-administered questionnaire was distributed through popular social media platforms among diabetics in the Saudi population. The survey questions involved demographic data; diabetes status including the time of diagnosis, current medications, and the latest HbA1c level; and an assessment of the knowledge about DKA through queries related to diagnostic criteria, definition, risk factors, symptoms, and preventive measures.

Results

Our study involved 400 participants, and 42.5% of them were able to correctly identify DKA as an emergency requiring immediate medical attention. Regarding the awareness of DKA's symptoms among the participants, 33.8% correctly identified excessive thirst as a key indicator, followed closely by frequent urination (31.8%), and the characteristic fruity breath odor (31.3%). As for the awareness of the participants of the causes of DKA, 33.8% correctly linked forgetting insulin injections to DKA development. Encouragingly, 39.8% of participants identified regular blood sugar monitoring as the most effective way to prevent DKA.

Conclusions

Most patients in our study demonstrated limited knowledge of DKA. However, a significant portion of them was able to identify it as an emergency. To prevent such events, raising awareness about DM and its complications may serve as the first step toward better outcomes in diabetic patients. We believe our findings can be used to devise quality-improving interventions in this field.

## Introduction

Diabetes mellitus (DM) is a persistent metabolic condition characterized by hyperglycemia due to inadequate insulin levels. It can be classified into two types; type 1 DM (T1DM), which is marked by a severe shortage or near absence of insulin, and type 2 DM (T2DM), which is mainly caused by insulin resistance and insufficient pancreatic insulin secretion [1,2]. Based on data from the World Health Organization, over 422 million adults worldwide had DM in 2014, and it is anticipated that this number will continue to grow [3]. The long-term consequences of DM encompass a spectrum of severe microvascular complications, including diabetic retinopathy, neuropathy, and renal disease, alongside substantial macrovascular implications, notably cardiovascular disease [4,5]. One of the most significant acute complications of DM is diabetic ketoacidosis (DKA) [6]. This condition occurs when cells cannot absorb glucose due to insulin deficiency, leading the liver to convert fat into ketones as an alternative fuel source. The excessive production of ketones results in their accumulation in the bloodstream, leading to acidemia [7].

DKA is associated with a wide range of clinical manifestations, with polyuria and polydipsia being among the earliest indicators [8]. A study at the King Abdulaziz University Hospital found that most patients had abdominal pain (83.3%) and vomiting (91.7%), with a notable subset also experiencing dyspnea (28%) and fever (31%). The primary cause identified in this cohort was the cessation of insulin therapy (86.6%), followed by concurrent infection, which was observed in 38% of cases [9]. Studies have shown that DKA is the leading cause of death in children and teenagers with T1DM, accounting for around 50% of all deaths in diabetic individuals under the age of 24 years [10,11]. It has been shown that raising awareness about the condition can potentially prevent 70% of DKA episodes [12]. This highlights the importance of providing education on DKA to individuals with DM and their caregivers.

Although DM has been widely studied, few studies in Saudi Arabia have explored the general public's awareness of DKA, particularly among individuals with DM. A cross-sectional study conducted in Riyadh, Saudi Arabia, involving 150 participants found that only 30% of the population had a good understanding of DKA symptoms, and only 6% were knowledgeable about DKA treatment [13]. A study from Al-Taif in western Saudi Arabia revealed that awareness levels among Saudi adults with diabetes were insufficient. The study involved a questionnaire-based cross-sectional survey of 100 randomly chosen participants with diabetes, ensuring diversity in age range and educational levels. Of the participants, 56% had T1DM and 44% had T2DM; only 11% engaged in vigorous sports activities. Additionally, only 62% reported adhering strictly to their DM medication. The study found that 54% of the participants identified DKA as a complication of DM. However, only 9% recognized infections as a risk factor for DKA, and 29% associated DKA with febrile illnesses. Of note, 50% believed that physical stress might be linked to DKA [14].

The above findings reveal a significant gap in the Saudi literature regarding the knowledge and practices of Saudi patients with diabetes when it comes to DKA. To address this gap and provide potential researchers with an essential source of information, this study aims to investigate the knowledge, attitudes, and practices of diabetes patients in the western and northern regions of Saudi Arabia.

## Materials and methods

Study design and setting

This observational, descriptive, cross-sectional study was approved by the Research Ethics Committee (REC) at the Hail University. The study was conducted in 2023 among diabetic patients residing in the western and northern regions of Saudi Arabia. An online self-administered questionnaire on a Google Form, consisting of close-ended questions, was used. The questionnaire underwent validation by a panel of diabetes experts and was distributed through popular social media platforms among the general public, including Telegram, WhatsApp, Twitter, and others. It was available to be accessed between April 14, 2023, and August 31, 2023, and was voluntarily filled out by individuals who agreed to be part of the study. The inclusion criteria were as follows: men and women with either type 1 or type 2 Diabetes, residing in the western and northern regions. Individuals without diabetes were excluded from the study. The final sample consisted of 400 participants. The confidentiality of the participants was ensured, as no personal information or identifiers were collected in the questionnaire.

Statistical analysis

The survey consisted of multiple-choice questions organized into two sections. The first section collected demographic data, such as sex, age, marital status, educational level, and employment status. The second section included questions regarding the participants' diabetic status, including the time of diagnosis, the medication used to control diabetes, follow-up tests such as HbA1c, and an assessment of their knowledge about DKA through questions related to diagnostic criteria, definition, risk factors, symptoms, and preventive measures. Data analysis was performed using SPSS Statistics version 20 (IBM Corp., Armonk, NY). Qualitative variables were classified according to the objectives of the study, and their frequencies and percentages were calculated. The awareness toward DKA among the study subjects was assessed using a 5-point scoring system; the correct responses were identified and assigned a score of 1 while the wrong or “I don`t know” responses were assigned a score of 0. For questions with multiple responses, each correct option was given 1 point. There were 18 queries in total, and the total awareness score was calculated by summing up the scores obtained for all 18 items; the scores ranged from 1 to 18, with higher scores indicating a better awareness of DKA. We employed 1\3 and 2\3 levels to determine the level of awareness; patients' scores were regarded as indicating poor awareness if the score was below 1\3, moderate awareness if it was from 1\3 to 2\3, and good awareness if the score was above 2\3.

## Results

A total of 407 participants filled in the questionnaire, but seven responses were excluded as the patient did not fill out the survey properly. The demographic features of the study participants are described in Table 1. Western region accounted for 56.8% of participants, and more than half (58%) were female. Approximately 47% were married and 39.2% were between the ages of 20 and 29 years. The majority of the respondents had a higher education (51.5%).

**Table 1:** Sociodemographic data (N=400)

Variables			Frequency	Percentage
					Geographical location	Western region		227	56.80%
Northern region		173	43.20%		
Gender	Male		168	42.00%
	Female		232	58.00%
Age, years	Less than 20		37	9.20%
	20-29		157	39.20%
	30-39		60	15.00%
	40-49		75	18.80%
	50 or above		71	17.80%
Marital status	Single		169	42.20%
	Married		188	47.00%
	Widowed		19	4.80%
	Divorced		24	6.00%
Education	Uneducated		47	11.80%
	Elementary school		20	5.00%
	Middle school		26	6.50%
	High school		66	16.50%
	Bachelor’s degree		206	51.50%
	Postgraduate		35	8.80%
Occupation	Student		73	18.20%
	Unemployed/in healthcare		109/53	27.3%/13.2%
	Not in healthcare		165	41.20%

As shown in Table 2, 36.5% of patients had been diagnosed with T1DM; 38.5% of the total participants stated that they had diabetes for 10 years or more. Additionally, 44.5% of participants were using insulin injections as monotherapy for their diabetes. Also, 68.8% were monitoring their Hb1Ac with a healthcare provider and 70% were monitoring their blood sugar at home.

**Table 2:** Specifics of the diabetic history of the respondents DKA: diabetic ketoacidosis

Question	Response	Percentage	Frequency
Type of diabetes mellitus	Type l	36.5%	146
	Type ll	34.8%	139
	Don't remember	28.7%	115
When did you get diagnosed with diabetes?	1-4 years ago	36.5%	146
	5-9 years ago	25.0%	100
	10 years ago, or more	38.5%	154
Medications used	Insulin injection	44.5%	178
	Oral anti-hyperglycemic drugs (monotherapy)	19.0%	76
	Multiple oral anti-hyperglycemic	5.2%	21
	Injection + oral anti-hyperglycemic	9.8%	39
	Take medications, but don't remember what they are	21.5%	86
Do you visit a physician to monitor Hb1Ac?	Yes	68.8%	275
	No	31.2%	125
Do you monitor blood sugar at home?	Yes	70.0%	280
	No	30.0%	120
Have you been diagnosed with DKA?	Have not been diagnosed with it before	58.5%	234
	Yes, once	23.2%	93
	Twice	11.0%	44
	More than twice	7.2%	29

The awareness assessment is demonstrated in Table 3. Of note, 42.5% of the participants correctly answered the query “DKA is considered to be an emergency complication of DM which requires an urgent intervention”. Also, roughly a quarter (25.8%) of the study participants answered that the glucose level in DKA is more than 200 mg/dL. Moreover, 33.8% thought that one of the major triggers of DKA was forgetting to take insulin injections. The most common symptoms of DKA were extreme thirst (33.8%), followed by frequent urination (31.8%), and having a fruity-smelling breath (31.3%). Also, 39.8% believed that regularly monitoring blood sugar levels daily is the best way to prevent DKA. Most of the participants answered "I don’t know" for several questions, which indicated a poor level of awareness.

**Table 3:** Awareness assessment

Question	Frequency		Percentage
Diabetic ketoacidosis is considered to be one of the following:			
An emergency complication of diabetes mellitus that needs immediate treatment and care in a hospital	170		42.50%
One of the problems caused by diabetes, it can be treated at home without going to the doctor	40		10%
Do not know	190		47.50%
The glucose level in diabetic ketoacidosis will be			
More than 200 mg/dL	103		25.80%
Less than 100 mg/dL	79		19.8
Do not know	218		54.50%
All these questions are variable with multiple-response answers			
What are the factors that may lead to diabetic ketoacidosis?			
Forgetting a dose of insulin	135		33.80%
Having type 1 diabetes, even if it has not been diagnosed	130		32.50%
Usage of certain drugs	79		19.80%
Heart disease, such as a heart attack	78		19.5
Being injured physically or emotionally	42		10.50%
Do not know	195		48.80%
What signs and symptoms do you think people with diabetic ketoacidosis might have?			
Extreme thirst	135		33.80%
Frequent urination	127		31.80%
The breath smells fruity	125		31.30%
Nausea and vomiting	117		29.3
Deep, rapid breathing	114		28.50%
Abdominal discomfort	101		25.30%
Feeling weak or tired	87		21.80%
Do not know	169		42.30%
How to prevent diabetic ketoacidosis?			
Monitor blood sugar levels daily regularly	159	39.80%	
Eat healthy food, exercise, and take medications	151	37.80%	
Adjust insulin dose as needed	127	31.80%	
Go to the emergency department when the blood sugar level is high	126	31.5	
Do not know	173	43.4	

**Figure 1:**
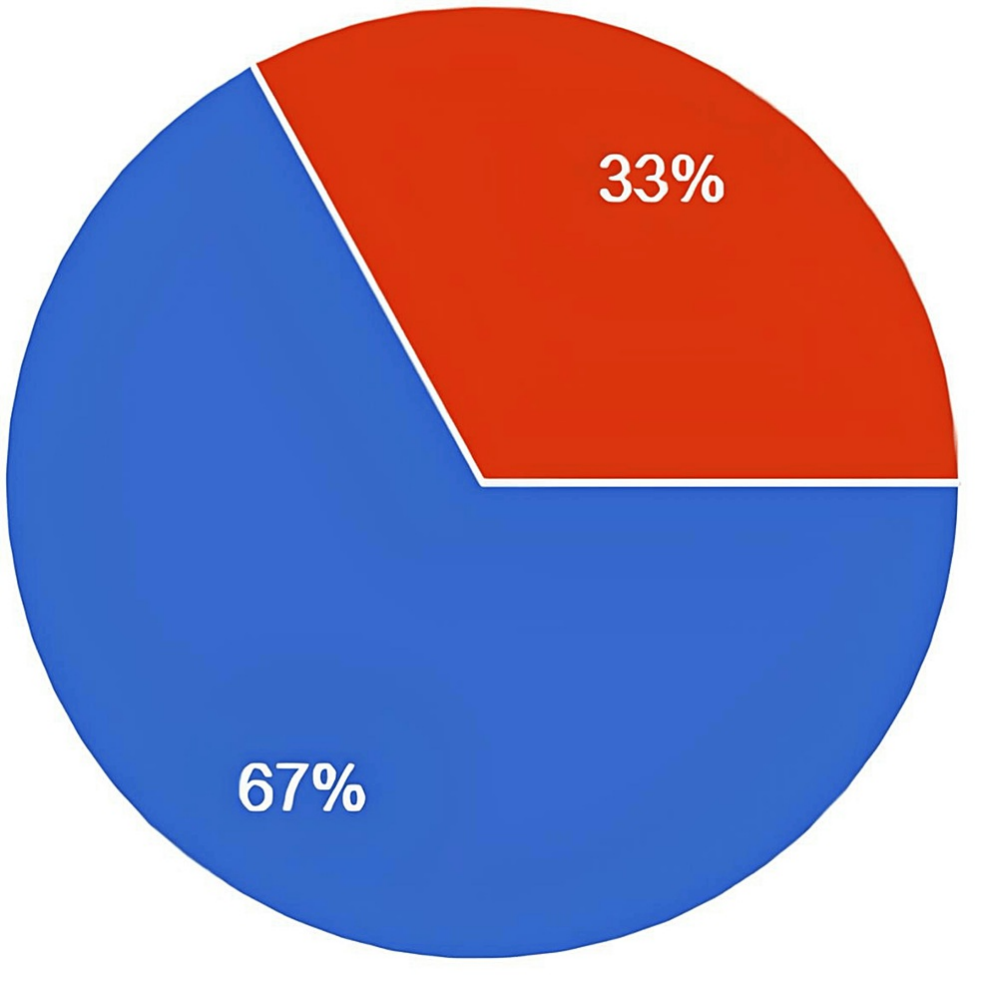
Do you believe you have adequate knowledge of diabetic ketoacidosis? Red: yes. Blue: no

**Figure 2:**
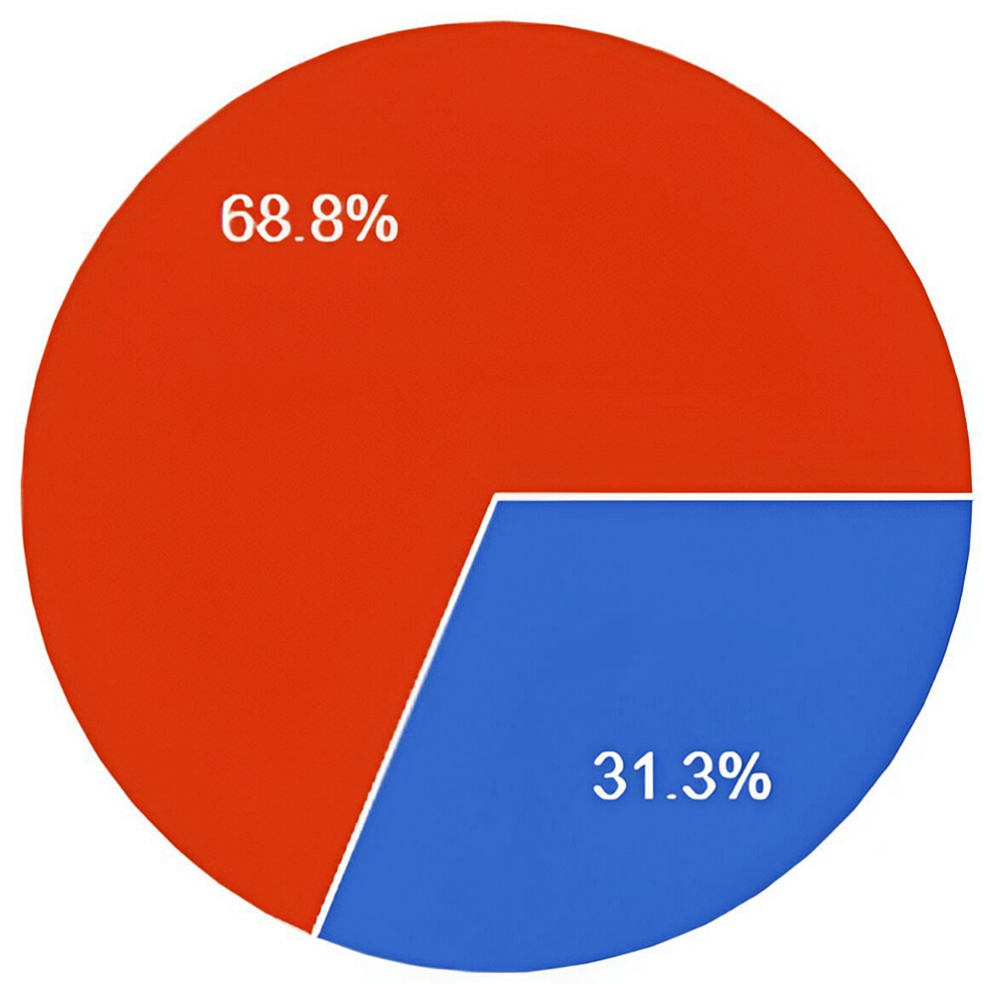
Do you believe that diabetic patients are not adequately educated about diabetic ketoacidosis? Red: yes. Blue: no

Significantly, two-thirds of the participants stated that they did not have adequate knowledge of DKA, as shown in Figures 1-2. Most diabetic patients believed that the community of diabetic patients was not well-educated about DKA.

**Figure 3:**
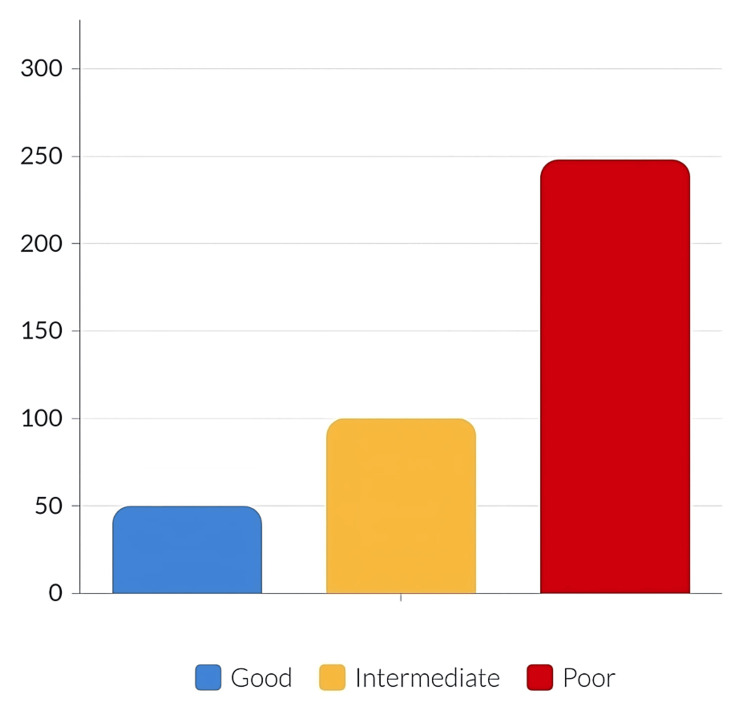
Level of awareness

The mean awareness score of the subjects was 5.405 (standard deviation: 5.18), and the total score was 18. Of note, 61.3% of the participants had poor awareness (score below or equal to 6/18), 25% of the participants had moderate awareness (score between 7 and 12/18, and 13.7% of the participants had a good awareness (score between 13 and 18/18) (Figure 3).

## Discussion

This study aimed to assess the knowledge of DKA and its related risk factors among diabetic patients. Approximately 47.5% of the participants lacked awareness of DKA, while 10% provided incorrect answers, indicating a significant deficit in knowledge regarding this life-threatening complication of DM. Also, our study revealed a higher lack of knowledge compared to previous studies conducted in Sudan and Riyadh [6-8] where 21.8%, 33%, and 35.3% of the participants respectively indicated a lack of awareness of DKA. Moreover, our study highlighted that 33.8% of participants believed that forgetting to take insulin injections was a leading cause of DKA, highlighting a serious misunderstanding of a major contributing risk factor. A similar study conducted in Sudan [6] showed that 39% held this belief, while another study from Saudi Arabia showed [14] that 32% believed that forgetting to take insulin injections is a risk factor, all indicating that these patients may develop DKA in the future. However, a study conducted in Riyadh revealed that 66% of participants were aware that failing to take insulin injections on time posed a risk for DKA. Their results could be attributed to the fact that the Riyadh region has a much better health and education system than other regions in Saudi Arabia. Additionally, the study demonstrated a significant knowledge gap and an urgent need for community education about medication compliance in other regions [13]. 

Exposure to high levels of cortisol caused by physical or psychological stress is known to increase the risk of developing DKA [15]. Interestingly, 89.5% of our participants disagreed with this statement, which is significantly higher than the 50% of participants in Alanazi et al.'s study who denied any correlation between stress and DKA [14]. The difference in findings between the studies indicates variations in the level of awareness among different populations and may be attributed to differences in healthcare systems, educational programs, and cultural factors. Regarding knowledge about signs and symptoms among our study participants, 33.8% reported that the most common symptom is extreme thirst while 31.8% stated that it is frequent urination; 42.3% answered "I don’t know" to this query. Unfortunately, the knowledge of other symptoms was low, and it was even lower than that in the Sudanese study [6], where 52% stated that extreme thirst was the most common symptom while 49.1% picked frequent urination. This variance could be attributed to the larger number of participants in our study compared to the Sudanese study. The study conducted in Riyadh [8] indicated that the most recognized symptoms of DKA were the characteristic smell of breath (70.0%) and vomiting (66.2%). The disparity in results might be because the researchers did not include extreme thirst and frequent urination as options.

Lastly, regarding knowledge about preventing DKA, 39.8% of participants believed that monitoring blood sugar levels regularly daily is the best preventive measure. Our assessment of the awareness of other prevention methods showed similar outcomes. This contrasts with the study conducted in Riyadh in 2021 [8], where 88.9% of the participants stated that DKA can be prevented by taking insulin as directed, while 88.4% mentioned continuous blood glucose monitoring. Again, these results could be attributed to the Riyadh region having a much better health and education system than other regions in KSA. About two-thirds of the participants in our study had a poor level of awareness of DKA. This is slightly higher than the study conducted in Sudan [6], which reported that 56.9% of the participants had a poor knowledge of DKA. Our values are also higher than those from a previous study in Saudi Arabia in 2018 [14], which showed that more than half of the respondents reported a lack of or low knowledge regarding DKA and its risk factors.

This highlights the need to raise awareness regularly about medical issues in the general population, especially among diabetics. The lack of health education and awareness and the public, parents, and primary healthcare physicians may be the cause of the lack of awareness of DKA. We recommend enhancing patient education for diabetic patients about DKA in diabetic clinics and raising public awareness about DKA through campaigns and on social media platforms.

Limitations of the study

This was a cross-sectional observational study, and a certain element of participation bias may be present, such as the discrepancy in participation rates between the western and northern regions, which could be attributed to the survey's distribution patterns and may reduce the precision of knowledge estimation. In addition, the online nature of the survey may have introduced an element of uncertainty among participants, leading them to search online for the correct responses to some of the survey's questions.

## Conclusions

Our study assessed participant’s knowledge of DKA in the northern and western regions of Saudi Arabia, and it revealed a low level of awareness, which is very concerning. Approximately 67% of survey responders believed that they did not have adequate knowledge about DKA and a significant proportion lacked understanding of its risk factors and symptoms. These findings stress the urgent need for comprehensive patient education initiatives within clinics and broader public awareness campaigns to address the knowledge deficit. The difference in knowledge levels across regions of KSA also underscores the importance of improving regional healthcare systems and addressing cultural factors in shaping health education. Closing these knowledge gaps is crucial for improving diabetes management and reducing the risk of life-threatening complications associated with DKA.

## Appendices

Questionnaire:

1-Gender:

A) Male

B) Female

2-Age in years:

A) 20-29

B) 30-39

C) 40-50 or more

D) Less than 20

3-Marital status:

A) Single

B) Married

C) Divorced

D) Widowed

4-Level of education:

A) Illiterate

B) Primary

C) Secondary

D) High school

E) University

F) Postgraduate education

5-Employment status:

A) Health-related (health specialization)

B) Non-health-related (non-health specialization)

C) Unemployed

D) Student

6- What type of diabetes have you been diagnosed with?

A) Type one

B) Type two

C) Don’t know

7-How long have you been diagnosed with diabetes?

A) Less than one year

B) In the past 1 to 4 years

C) In the past 5 to 9 years

D) In the past 10 years and more

8-What medicines do take to control your blood sugar (you can choose more than one option):

A) Insulin injection

B) Sulphonylurea (diamicron, Amaryl)

C) Metformin

D) DD4 inhibitors (sitagliptin, Galvus)

E) Sodium-glucose transporters 2 inhibitors (dapagliflozin, empagliflozin)

F) I use tablets, but I don’t know their names

9- Are you visiting a doctor and checking your HBA1c regularly?

A) Yes

B) No

10- Do you check your blood glucose at home?

A) Yes

B) No

11- Have you been diagnosed with diabetic ketoacidosis?

A) Yes once

B) Two times

C) More than 2 times

D) Not diagnosed with DKA before

12-Do you believe that diabetic patients are not adequately educated about diabetic ketoacidosis?

A) Yes

B) No

13-Do you believe you have adequate knowledge of diabetic ketoacidosis?

A) Yes

B) No

14- Diabetic ketoacidosis is considered to be one of the following:

A) An emergency complication of diabetes mellitus that needs immediate treatment and care in the hospital

B) One of the problems caused by diabetes, it can be treated at home without going to the doctor

C) Do not know

15- Glucose level in diabetic ketoacidosis will be:

A) More than 200 milligrams per deciliter

B) Less than 100 milligrams per deciliter

C) Do not know

16- What are the factors that may lead to diabetic ketoacidosis:

A) Heart disease, such as a heart attack

B) Forgetting a dose of insulin

C) Having type 1 diabetes, even if it has not been diagnosed

D) Usage of certain drugs

E) Being injured physically or emotionally

F) Infection

G) I don’t know

17- What signs and symptoms do you think people with Diabetic ketoacidosis might have? (You can choose more than one option):

A) Extreme thirst

B) Frequent urination

C) Nausea and vomiting

D) Deep, rapid breathing

E) Abdominal discomfort

F) The breath smells fruity

G) Feeling weak or tired

H) I Don’t know

18- How to Prevent Diabetic Ketoacidosis?

A) Eat healthy food, exercise, and take medication

B) Monitor blood sugar level daily regularly

C) Adjusting insulin dose as needed

D) Go to the emergency department when the blood sugar level is high

E) I don’t know
